# Increased Expression of Interleukin-18 mRNA is Associated with Carotid Artery Stenosis

**DOI:** 10.4274/balkanmedj.2017.0323

**Published:** 2018-05-29

**Authors:** Berk Arapi, Burcu Bayoğlu, Müjgan Cengiz, Ahmet Dirican, Serkan Burç Deser, Yerik Junusbekov, Caner Arslan

**Affiliations:** 1 Department of Cardiovascular Surgery, İstanbul University Cerrahpaşa School of Medicine, İstanbul, Turkey; 2 Department of Medical Biology, İstanbul University Cerrahpaşa School of Medicine, İstanbul, Turkey; 3 Department of Biostatistics and Medical Informatics, İstanbul University İstanbul School of Medicine, İstanbul, Turkey; 4 Department of Cardiovascular Surgery, Ondokuz Mayıs University School of Medicine, İstanbul, Turkey

**Keywords:** Carotid artery, gene expression, interleukin-18, interleukin-18-binding protein, stenosis

## Abstract

**Background:**

Carotid artery stenosis is the atherosclerotic narrowing of the proximal internal carotid artery and one of the primary causes of stroke. Elevated expression of the pleiotropic proinflammatory cytokine interleukin-18 has been demonstrated in human atherosclerotic plaques.

**Aims:**

To investigate whether the mRNA expression levels of interleukin-18 and interleukin-18-binding protein and interleukin-18 −137 G/C (rs187238) variants are associated with carotid artery stenosis development.

**Study Design:**

Case-control study.

**Methods:**

The mRNA expression levels of interleukin-18 and interleukin-18-binding protein and interleukin-18 rs187238 variants were evaluated by quantitative real-time polymerase chain reaction and real-time polymerase chain reaction, respectively, in the peripheral blood mononuclear cells of 70 patients with carotid artery stenosis (36 symptomatic, 34 asymptomatic) and 75 healthy controls.

**Results:**

Interleukin-18 mRNA expression was significantly increased in carotid artery stenosis patients compared to that in healthy controls (p=0.01). However, no significant difference was observed between interleukin-18-binding protein mRNA expression levels in patients with carotid artery stenosis and those in controls (p=0.101). Internal carotid artery stenosis severity was significantly higher in symptomatic patients than that in asymptomatic patients (p<0.001). A significant relationship was identified between interleukin-18 expression and internal carotid artery stenosis severity in patients with carotid artery stenosis (p=0.051). Interleukin-18 rs187238 polymorphism genotype frequencies did not significantly differ between patients with carotid artery stenosis and controls (p=0.246). A significant difference was identified between interleukin-18-binding protein gene expression and symptomatic and asymptomatic patients (p=0.026), but there was no difference in interleukin-18 expression between the symptomatic and asymptomatic subgroups (p=0.397).

**Conclusion:**

Interleukin-18 mRNA expression may affect carotid artery stenosis etiopathogenesis and internal carotid artery stenosis severity and also may play a mechanistic role in the pathogenesis of carotid artery stenosis, influencing the appearance of symptoms.

The primary cause of cardiovascular morbidity and mortality is atherosclerosis. Carotid artery stenosis (CAS) usually occurs proximally in the bulbous internal carotid artery (ICA). Several factors play crucial roles in CAS pathogenesis, including environmental and genetic. Thus, it has a multifactorial and polygenic inheritance pattern. Canonical risk factors for cardiovascular diseases (CVDs), including age, sex, smoking status, dyslipidemia, and diabetes with associated biochemical risk factors, lipoprotein (a), fibrinogen, homocysteine, and C-reactive protein (CRP), may be related to the molecular pathophysiology contributing to advanced CAS. A series of inflammatory events are involved in the initiation and progression of atherosclerosis. In addition, carotid plaque formation has been reported to be a subclinical ultrasonographic marker of CVD and stroke (1). Several studies have shown variants of genes encoding cytokines and chemokines, as well as their receptors, to be associated with CVD risk (2,3).

The induction, progression, and the rupture of atherosclerotic lesions have been closely associated with inflammatory mediators (4). Cytokines such as interleukin (IL)-12, interferon-gamma (IFN-γ), and *IL-18* have essential roles in the formation and progression of atherosclerotic plaques. *IL-18*, also known as IFN-γ-inducing factor, was determined in Kupffer cells and macrophages (5). In addition, *IL-18* has been shown to have proinflammatory, proapoptotic, and proatherogenic activities in diabetes and CVD (6,7). *IL-18* expression is tissue-specific, which depends on its promoter region (8,9). Furthermore, patients with acute myocardial infarction (AMI) have been found to have augmented *IL-18* levels (10). A study reported that *IL-18* blockage is associated with diminished atherosclerotic lesion formation and advancement in experimentally *ApoE*-deficient mice (11). Moreover, *IL-18* levels are found to be 3-fold higher in unstable lesions than in stable plaques. Furthermore, repression of *IL-18* has been shown to have positive effects on lesion composition and progression (12). Therefore, *IL-18* is a significant agent of atherosclerotic lesion destabilization and vulnerability. *IL-18*-binding protein (*IL-18-BP*) balances *IL-18* activity through high-affinity binding with *IL-18*, which prevents *IL-18* from binding to its receptor. Improved intensity of atherosclerosis may be related to changes in *IL-18*/*IL-18-BP* balance, and elevated free *IL-18* levels have been identified in the circulation of disease states (13,14,15,16).

Human *IL-18* rs187238 has allelic variations at the −137 position of the promoter region resulting in a guanine-to-cytosine (G/C) substitution. Rs187238 has been reported to be in complete linkage disequilibrium with two additional variations, at position +113 and +127, and regulates *IL-18* production in mononuclear cells (17). Rs187238 variants also influence *IL-18* transcriptional activity (17,18,19). In addition, circulating *IL-18* levels showed a positive correlation with carotid intima-media thickness (20). To our knowledge, peripheral blood mononuclear cell (PBMC)-specific mRNA levels of *IL-18* and *IL-18-BP* in patients with CAS and the relationship with SNP rs187238 have not yet been investigated. Given that inflammation is a significant risk factor for atherosclerosis and the associated macrovascular and microvascular complications, we hypothesized that the mRNA expression of *IL-18* and *IL-18-BP*, along with rs187238 SNP, may have a significant role in CAS development. Therefore, we explored whether *IL-18* and *IL-18-BP* expression and *IL-18* rs187238 −137 G/C variants were associated with CAS.

## MATERIALS AND METHODS

### Study group

The study group comprised a total of 145 Turkish individuals, including 70 subjects with CAS (36 symptomatic, 34 asymptomatic) and 75 healthy controls. *IL-18* and *IL-18-BP* expression and rs187238 variants were analyzed in patients diagnosed with CAS and in healthy individuals. Patients diagnosed with CAS and controls were selected from the İstanbul University, Cerrahpaşa School of Medicine, Department of Cardiovascular Surgery. This research was performed in agreement with the principles of the Declaration of Helsinki and was confirmed by İstanbul University, Cerrahpaşa School of Medicine local ethics committee, 6 November 2012, No: 33275. Written informed permission was collected from all subjects prior to the commencement of the study. Patients with ≥70% atherosclerotic stenosis of carotid arteries as detected by color Doppler ultrasonography, computed tomography-angiography, magnetic resonance angiography, and digital subtraction angiography were included. All subjects with CAS (age range: 46-83 years), including 36 symptomatic and 34 asymptomatic, underwent carotid endarterectomy surgery in our department. According to the classification, patients with ≥70% stenosis of the ICA and cerebrovascular episodes of stroke, transient ischemic attack, or amaurosis fugax prior to carotid artery examination were selected as symptomatic. Asymptomatic patients were those with ICA stenosis of ≥70% but without clinical symptoms of CAS. All subjects in the patient group were taking a statin treatment.

A total of 75 healthy individuals (age range: 44-85 years) who visited the hospital for usual health screenings were selected as controls. Inclusion criteria for the control group were a normal lipid profile and lack of statin use. Exclusion criteria were any inflammatory disease, CVD, diabetes, hypertension, severe kidney and hepatic diseases, autoimmune disease, cancer, and pregnancy.

### Collection of blood specimens and genomic DNA purification

Venous blood samples from patients with CAS and healthy donors were collected in standard K3 EDTA-containing tubes. Genomic DNA extraction from whole blood was performed using a commercial kit (Roche Diagnostics GmbH, Mannheim, Germany).

### Collection of blood specimens, PBMCs, and total RNA extraction

Venous blood specimens were collected into Na-heparin tubes and used for lymphocyte isolation and RNA purification as previously described (21).

### Reverse transcription-quantitative real-time polymerase chain reaction

DNase I treatment (Invitrogen, Carlsbad, California, USA) was performed during total RNA extraction. RNA concentration was evaluated using a Nanodrop Spectrophotometer (ThermoScientific, Waltham, MA, USA). Total RNA (400 ng) was reverse-transcribed as previously described (21). The mRNA levels IL18 and *IL-18-BP *in PBMCs were determined by reverse transcription-quantitative real-time polymerase chain reaction (RT-qPCR) using Universal Probe Library probes, the TaqMan method, and a LightCycler 1.5^®^ instrument (Roche Applied Biosystems^TM^, California, USA). The expression levels of *IL-18* and *IL-18-BP* were normalized against those of ACTB and B2M endogenous references. Negative controls, without a cDNA template, were included in each run. Ct values of 40 were not included in the research. The experiments were conducted in duplicate.

### *IL-18* −137 G/C (rs187238) variants

Rs187238 *IL-18* variants were established by RT-PCR using 3ʹ-fluorescein and 5ʹ-LightCycler^®^ Red labeled hybridization probes (TIB MOLBIOL GmbH, Berlin, Germany). PCR was performed using 20-μL volume containing 2.0 μL of master mix (Roche Diagnostics GmbH), 1.0 μL Reagent mix, 3.0 mM MgCl_2_, and 50 ngDNA. To assess the eligibility of genotyping, the patient and control samples were randomly selected for independent replication of genotyping and repeated and the initial results were compared.

### Statistical analysis

Statistical analyses were carried out using Student’s t-test to compare the values obtained for patients and controls. Data were represented as mean ± standard deviation. Categorical variables were analyzed using the chi-square (χ2) test. Case-control comparisons between genotypes and alleles and deviations from the Hardy–Weinberg equilibrium (HWE) were compared by χ2 test. The threshold value for the statistical significance was taken as p≤0.05. The reliability of the relationship between the genotypes, alleles, and case-control comparisons was established by calculating the odds ratios and 95% confidence intervals. Data were analyzed under a dominant model. Ct values were used to calculate relative target mRNA levels using the 2^-∆Ct^ formula. Results were analyzed by Student’s t-test. All the data were analyzed using SPSS software for Windows (Version 21.0) (IBM Corp, NY, USA).

## RESULTS

### Demographic data

No statistically significant differences were detected between patients with CAS and control subjects in terms of age (p=0.237) and gender (p=0.813). Significant differences were observed between patients with CAS and control subjects in terms of LDL-cholesterol (p=0.005), fasting glucose (p<0.001), urea (p=0.001), creatinine (p=0.002), and CRP (p<0.001) levels. However, regarding the levels of triglycerides, hematocrit, aspartate aminotransferase (AST), alanine aminotransferase (ALT), total cholesterol, and high-density lipoprotein (HDL) cholesterol, there were no significant differences between patients with CAS and control subjects (p>0.05) (Table 1).

There were also no significant differences between symptomatic and asymptomatic patients with CAS in terms of age, sex, total cholesterol, HDL-cholesterol, low-density lipoprotein (LDL) cholesterol, triglycerides, fasting glucose, hematocrit, urea, creatinine, AST, ALT, or CRP levels (p>0.05). Eligible patients were recruited based on having ≥70% atherosclerotic stenosis of the carotid arteries. Since CAS is primarily treated using pharmacotherapy in asymptomatic patients with <80% stenosis, we further divided the subjects with CAS into two groups, those with <80% and those with ≥80% stenosis. The degree of ICA stenosis severity was significantly augmented in symptomatic patients when compared to that in asymptomatic patients (p<0.001). Moreover, a borderline significant difference in the incidence of hypertension was observed between symptomatic and asymptomatic patients (p=0.055). No significant differences were detected in the prevalence of type 2 diabetes, peripheral artery disease, and coronary artery disease (CAD) between symptomatic and asymptomatic patients with CAS (p>0.05) (Table 2). There were also no significant differences in total-, LDL-, and HDL-cholesterol, triglycerides, fasting glucose, hematocrit, urea, creatinine, ALT, AST, and CRP levels between patients with <80% and those with ≥80% ICA stenosis (p>0.05) (data not shown).

### mRNA levels of *IL-18* and *IL-18-BP*

**Figure 1:**
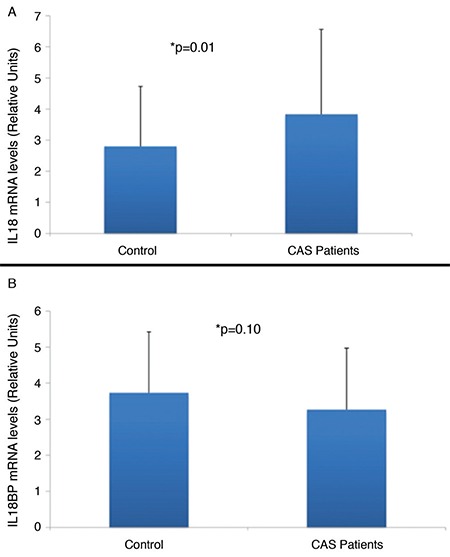
Relative IL-18 and IL-18-BP mRNA expressions in patients with carotid artery stenosis and healthy controls. *CAS: carotid artery stenosis; IL-18: interleukin-18; IL-18-BP: interleukin-18-binding protein*

The expression of *IL-18* was significantly elevated in subjects with CAS compared to that in healthy controls (p=0.01). Nevertheless, no statistical differences in *IL-18-BP* expression were found between patients with CAS and controls (p=0.10) (Figure 1). There was no significant association between *IL-18* mRNA levels and patients with symptomatic and asymptomatic CAS (t=−0.85, p=0.397). However, a significant relationship was observed between *IL-18-BP* expression and patients with symptomatic and asymptomatic CAS (t=2.27, p=0.026). In addition, an almost statistically significant relationship was identified between *IL-18* gene expression and the degree of ICA stenosis (p=0.051).

### Frequencies of *IL-18* −137 G/C (rs187238) variants

*IL-18* −137 G/C (rs187238) genotype distribution conformed to HWE expectations among patients with CAS (χ2=2.09, p=0.148) and controls (χ2=0.79, p=0.37). There were no statistically significant differences in rs187238 variants between patients with CAS and the control group (p=0.246). Furthermore, no significant differences were observed in rs187238 allele frequencies between the patient and control groups (p=0.53) (Table 3). Moreover, *IL-18* rs187238 genotype and allele frequencies in symptomatic and asymptomatic subgroups of patients with CAS showed no significant differences (p>0.05) (data not shown).

## DISCUSSION

This study was performed to explore the associations between *IL-18* and *IL-18-BP* gene expression levels, the *IL-18* rs187238 variants, and the development of CAS. Altogether, the results of this study suggest that *IL-18* expression may serve as a marker for the genetic predisposition of CAS, whereas *IL-18* gene −137 G/C variants do not influence CAS pathogenesis.

Some studies have reported the presence of serum *IL-18* levels, *IL-18* gene variants, and mRNA levels in AMI, CAD, stroke, and atherosclerosis (12,22,23,24,25). In addition, *IL-18* −137 G/C variants have been shown to regulate circulating *IL-18* levels (18,19). However, to our knowledge, this is the first investigation to examine the relationship between PBMC *IL-18* and *IL-18-BP* expression and the effect of rs187238 variants in symptomatic and asymptomatic subjects with CAS.

*IL-18* protein levels were reported to be increased in human atheroma plaques, and in carotid plaques, especially in macrophages (24). Furthermore, in severe CAS, increased *IL-18* mRNA levels have been identified in unstable plaques (12). Resident inflammatory macrophages and foam cells make plaques more vulnerable to rupture. Therefore, reducing the activity of these cells is important for the prognosis of atherosclerosis (26). In our study, LDL-cholesterol, fasting glucose, urea, creatinine, and CRP levels showed significant alterations between CAS and control groups. Significantly elevated *IL-18* expression in the PBMCs of CAS group indicates a critical link between *IL-18* gene expression, inflammation, and CAS pathogenesis. To date, a correlation between CAS and *IL-18* gene expression in PBMCs has not been addressed. Our results are consistent with those of previous studies describing a relationship between atherosclerotic plaque tissue expression and *IL-18* levels. However, we detected no significant difference in *IL-18* levels between symptomatic and asymptomatic subjects with CAS. This result is also inconsistent with that of a previous study (12) and may be due to the small subgroup sample size in this study. Chang et al. (27) concluded that elevated serum *IL-18* levels could be a marker of cardiocerebral events in dialysis patients. In our study, *IL-18* mRNA expression did not differ between symptomatic and asymptomatic patient groups. However, a borderline significant relationship was observed between ICA stenosis severity and *IL-18* mRNA expression. We observed several critical differences in the demographics and clinical characteristics of symptomatic and asymptomatic CAS subgroups. Although elevated CRP levels were observed in patients with severe ICA stenosis, this result was not statistically significant.

Recently, *IL-18-BP* was identified as a Na-Cl cotransporter associated with *IL-18*R and essential for *IL-18*-mediated signaling and for promoting experimental atherogenesis (28). In this study, *IL-18-BP* expression was not significantly associated with CAS development, suggesting that *IL-18-BP* did not inhibit *IL-18* binding to its receptor and the subsequent proinflammatory signals in patients with CAS. Thus, *IL-18* expression was augmented in our CAS group.

Recently, we showed that *IL-18* expression was higher in patients with peripheral artery disease (PAD) than that in healthy controls; nonetheless, this finding did not achieve a statistically significant level. In addition, *IL-18-BP* gene expression diminished significantly in patients with PAD compared to that in healthy subjects (29). These results were also consistent with our previous findings, suggesting a significant relationship between *IL-18*, related genes, and CAS pathogenesis.

A significant association has been identified between −607 C/A and AMI (23), and the −137 G/C polymorphism has been associated with CAD and affects serum *IL-18* levels. Furthermore, the *IL-18* −137 G/C variation G allele frequency has been shown to be elevated in patients with CAD, as have *IL-18* protein levels (22). *IL-18* gene −607 C/A and −137 G/C variants were compared between subjects having had ischemic stroke and healthy controls in a Han Chinese cohort. The −607C variant was found to correlate with the risk of ischemic stroke, while the −137G variant was related to an elevated risk of large artery stenosis (23). In our study, *IL-18* −137 G/C genotype and allele frequencies were not associated with CAS. In addition, *IL-18* −137 G/C variation genotype and allele frequencies did not significantly differ between symptomatic and asymptomatic patients with CAS. Our findings are not parallel with those of previous reports examining atherosclerotic occlusive disease. This discrepancy may indicate that different molecular mechanisms function in the pathogenesis of carotid stenosis, a cerebrovascular disease that, along with ethnic differences, may also play a role in the susceptibility to CAS.

A proinflammatory role of *IL-18* in atherogenesis is supported by observations that *IL-18* and its receptor are expressed on human atheroma-associated endothelial cells, smooth muscle cells (SMCs), and mononuclear phagocytes and that *IL-18* can induce IFN-γ expression in SMCs (24). Comparison of early subclinical markers of atherosclerosis in healthy young adults revealed that the carotid artery intima-media ratio was not associated with *IL-18* gene variants (25). In addition, it has been shown that elevated serum *IL-18* levels mildly influence CAD development in healthy males (30). In our study, *IL-18* expression showed no significant gender difference in subjects with CAS, suggesting that *IL-18* transcription may not have a gender-specific impact on CAS.

CAS is a complex disease with multifactorial and polygenic inheritance, and genetic and environmental factors may influence its pathogenesis. This investigation recommends that *IL-18* gene expression can be an important marker for ICA stenosis. However, this investigation has some limitations, along with its limited sample size. Further research is required to fully elucidate the role of *IL-18* and its genetically related pathways in vascular occlusive diseases. In the future, *IL-18* may be included in the progression of preventive therapies to reduce the incidence of cerebrovascular diseases.

## Figures and Tables

**Table 1:**
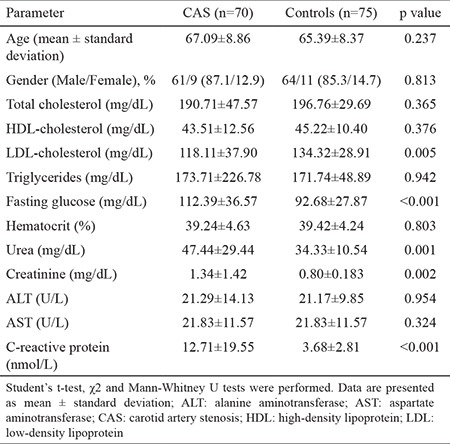
Baseline demographics and clinical characteristics of patients with carotid artery stenosis and controls

**Table 2:**
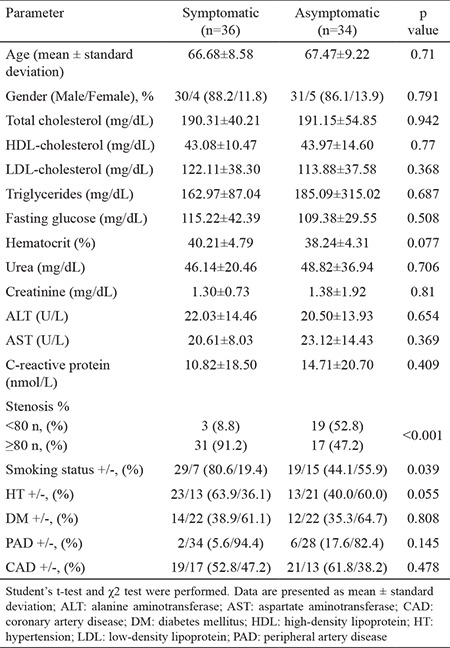
Demographics and clinical characteristics of patients with symptomatic and asymptomatic carotid artery stenosis

**Table 3:**
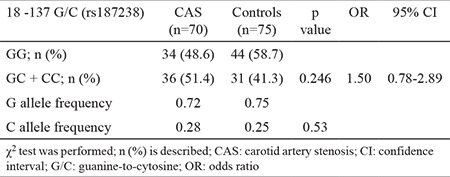
Distribution of IL-18 -−137 G/C (rs187238) polymorphism genotype and allele frequencies in patients with carotid artery stenosis and healthy controls
